# Identification of lipid metabolism-related biomarkers for diagnosis and molecular classification of atherosclerosis

**DOI:** 10.1186/s12944-023-01864-6

**Published:** 2023-07-06

**Authors:** Xue Pan, Jifeng Liu, Lei Zhong, Yunshu Zhang, Chaosheng Liu, Jing Gao, Min Pang

**Affiliations:** 1 Liaoning University of Traditional Chinese Medicine, Shenyang, Liaoning China; 2 Dazhou Vocational College of Chinese Medicine, Dazhou, Sichuan China; 3 The First Affiliated Hospital of Dalian Medical University, Dalian, Liaoning China; 4 The Second Affiliated Hospital of Liaoning University of Traditional Chinese Medicine, Shenyang, Liaoning China

**Keywords:** Atherosclerosis, Lipid metabolism, Immune infiltration, Machine learning, Biomarkers, Molecular clusters

## Abstract

**Background:**

Atherosclerosis is now the main cause of cardiac-cerebral vascular diseases around the world. Disturbances in lipid metabolism have an essential role in the development and progression of atherosclerosis. Thus, we aimed to investigate lipid metabolism-related molecular clusters and develop a diagnostic model for atherosclerosis.

**Methods:**

First, we used the GSE100927 and GSE43292 datasets to screen differentially expressed lipid metabolism-related genes (LMRGs). Subsequent enrichment analysis of these key genes was performed using the Metascape database. Using 101 atherosclerosis samples, we investigated the LMRG-based molecular clusters and the corresponding immune cell infiltration. After that, a diagnostic model for atherosclerosis was constructed using the least absolute shrinkage and selection operator (LASSO) and multivariate logistic regression. Finally, a series of bioinformatics techniques, including CIBERSORT, gene set variation analysis, and single-cell data analysis, were used to analyze the potential mechanisms of the model genes in atherosclerosis.

**Results:**

A total of 29 LMRGs were found to be differentially expressed between atherosclerosis and normal samples. Functional and DisGeNET enrichment analyses indicated that 29 LMRGs are primarily engaged in cholesterol and lipid metabolism, the PPAR signaling pathway, and regulation of the inflammatory response and are also closely associated with atherosclerotic lesions. Two LMRG-related molecular clusters with significant biological functional differences are defined in atherosclerosis. A three-gene diagnostic model containing ADCY7, SCD, and CD36 was subsequently constructed. Receiver operating characteristic curves, decision curves, and an external validation dataset showed that our model exhibits good predictive performance. In addition, three model genes were found to be closely associated with immune cell infiltration, especially macrophage infiltration.

**Conclusions:**

Our study comprehensively highlighted the intricate association between lipid metabolism and atherosclerosis and created a three-gene model for future clinical diagnosis.

**Supplementary Information:**

The online version contains supplementary material available at 10.1186/s12944-023-01864-6.

## Introduction

Cardiac-cerebral vascular disorders (CCVDs) pose a severe hazard to human health, especially for the population over the age of 50 [1]. One of the world’s biggest causes of death, CCVDs, which are predominantly caused by atherosclerosis (AS), claim 15 million lives annually [2]. Endothelial dysfunction is the beginning of the complex inflammatory disease AS, which progresses to plaque production, instability, and rupture after aberrant immune and tissue repair responses [3]. Due to the progressive nature of atherosclerotic plaque formation and the fact that many risk factors are modifiable, there is a window of opportunity for presymptomatic recognition. However, intervention is usually not performed until symptoms occur, even at the onset of CCVD. Therefore, there is an urgent need to develop advanced molecular biomarkers for early diagnosis of AS.

The function of lipids has drawn more attention over the lengthy history of research on AS. The findings of multiple investigations demonstrate the various roles played by lipids in atherogenesis. Lipids are essential components of biological membranes and other cell structures, including phospholipids, fatty acids, triglycerides, sphingolipids, cholesterol, and cholesteryl esters [4]. Modern theories view AS as a condition marked by an excessive buildup of lipids in the artery wall and a disturbed balance between the systems involved in the onset and resolution of inflammation. It has also been suggested that AS begins with the accumulation of Apo B-containing lipoproteins in the arterial intima, accompanied by the activation of endothelial cells and the recruitment of leukocytes, especially monocytes, and leads to the accumulation of cells, extracellular matrix and lipids in the arterial intima [5]. Meanwhile, targeting lipid metabolism is a major approach to managing and preventing AS [6]. Thus, it is promising to explore the molecular subtypes of AS patients based on lipid metabolism-related genes (LMRGs) and to construct new diagnostic biomarkers.

In this study, we conducted the first systematic examination of the differentially expressed LMRGs and immunological features between normal and AS patients. Next, 101 AS patients were separated into two lipid metabolism-related clusters based on the 29 DE-LMRG expression landscapes, and immune cell infiltration and crucial pathway differences between the two groups were examined further. Three LMRGs (ADCY7, CD36, and SCD) were discovered as diagnostic models by applying the least absolute shrinkage and selection operator (LASSO) and multivariable logistic regression. To validate the diagnostic model’s efficacy, receiver operating characteristic (ROC) curve decision curve analysis (DCA) and external sets were utilized. Finally, we analyzed the biological characteristics of the three model genes and explored their correlation with immune cells. These results enhance our understanding of the mechanism of lipid metabolism and provide new strategies for the early diagnosis of AS.

## Methods

### Data preparation

Three datasets (GSE100927 [7], GSE43292 [8], and GSE28829 [9]) related to AS were obtained from the GEO database. Then, the GSE100927 and GSE43292 datasets were combined as a training set for further analysis. The combat technique from the “SVA” package was utilized to rectify batch effects [10]. Meanwhile, the GSE28829 dataset was selected as the test cohort. The LMRGs were obtained from the Molecular Signature Database (MSigDB) [11]. The LMRG was further filtered by intersecting the differentially expressed genes (DEGs) of GSE100927 and GSE43292. DEGs between AS and normal samples were identified using the “limma” package with adjusted *P* < 0.05 and FC > 1.5 as criteria [12].

### Analysis of functional enrichment

A comprehensive source for functional genomics is the Gene Ontology (GO) project [13]. The KEGG database, which integrates genomic, chemical, and systemic functional information, is also commonly utilized when studying biological pathway data [14]. Using Metascape, a web-based portal created to offer an extensive gene list annotation and analysis resource for experimental biologists, functional and DisGeNET enrichment studies were carried out to examine the biological functions and pathways implicated in DE-LMRGs [15].

### Unsupervised clustering of AS patients

We used the “ConsensusClusterPlus” R package to perform an unsupervised clustering analysis based on the expression profiles of DE-LMRGs [16], and we used the k-means technique with 1000 iterations to divide the AS samples into several groups. We set k to 9 and evaluated the appropriate cluster number based on the CDF curve, consensus matrix, and consistent cluster score.

### Construction of the prognostic model based on DE-LMRGs

To avoid overfitting, we used a LASSO regression analysis with the “glmnet” package, simplifying our model’s parameter [17]. We selected the relative parameters whose p values were less than 0.05 as the final parameters of the prediction model after a multivariable logistic regression analysis of the LASSO regression-produced influencing factors using the “glm” function. The risk score was determined by multiplying each LMRG expression level (α) by a linear combination of the corresponding coefficients (β). The area under the curve (AUC) of each receiver operating characteristic (ROC) curve was determined by applying the pROC package to R software to assess the model’s prediction accuracy [18]. Decision curve analysis (DCA), a novel tool, can be used to examine the applicability of this model on clinical net benefit under various positive thresholds [19]. A nomogram assessing the prevalence of AS was developed using the “rms” R package [20]. Each predictor has a corresponding score, and the “total score” is the result of adding all of the aforementioned predictors’ values together. To calculate the nomogram model’s predictive power, a calibration curve was used.

### Independent validation analysis

In addition, the GSE28829 dataset was used to validate the performance of the risk model to predict AS. The differential expression of three model genes in AS and normal tissues was compared using the “limma” package. In addition, ROC, DCA, and calibration curves were constructed to verify the performance of the model in the validation set.

### Immune Infiltration Analysis

The CIBERSORT algorithm and LM22 signature matrix were used to estimate the relative abundances of 22 different types of immune cells in each sample based on the prior gene expression data. An inverse fold product *P* value for each sample was calculated by CIBERSORT using Monte Carlo sampling. The definition of the accurate immune cell fraction was limited to samples with *P* values < 0.05. The sum of the 22 immune cell proportions in each sample was 1[21]. The Wilcoxon test was used to analyze the differences in immune cells between different groups.

### Gene set variation analysis (GSVA) and immune correlation analysis

GSVA enrichment analysis of each model gene was performed using the “GSVA” R package [22]. Considered considerably altered if the |t| value of the GSVA score was greater than 2. The relationship between each model gene and immunochemical was assessed and plotted based on CIBERSORT results. When the *P* value was less than 0.05, the Spearman correlation coefficient found that there was a significant link. Finally, the findings were shown with the “corrplot” R tool.

### Single-cell data analysis

We downloaded the GSE159677 dataset [23] for further analysis of model genes. The scRNA-seq data were processed with the R package “Seurat” [24], including filtered cells and genes, t-distributed stochastic neighbor embedding (t-SNE) and principal component analysis (PCA). Detection of marker genes for each cell cluster was performed by the “FindAllMarkers” function of the R package “Seurat”. The R package “SingleR” [25] was applied to annotate the cell types in different cell clusters. The R package “CellChat“[26] was used for cell‒cell interaction analysis.

## Results

### Identification of AS DEGs

**Fig. 1:**
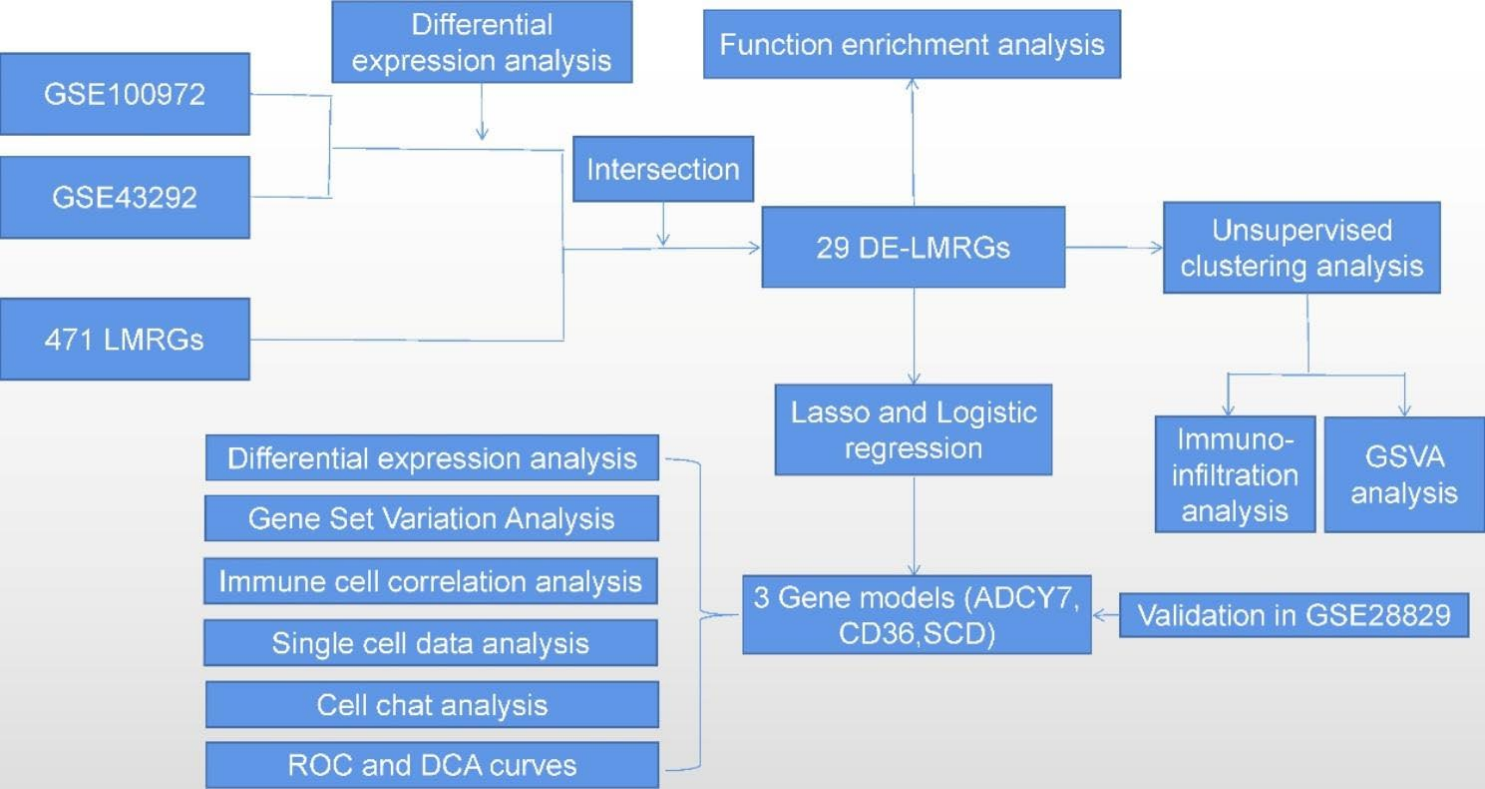
Flow chart of this study

**Fig. 2:**
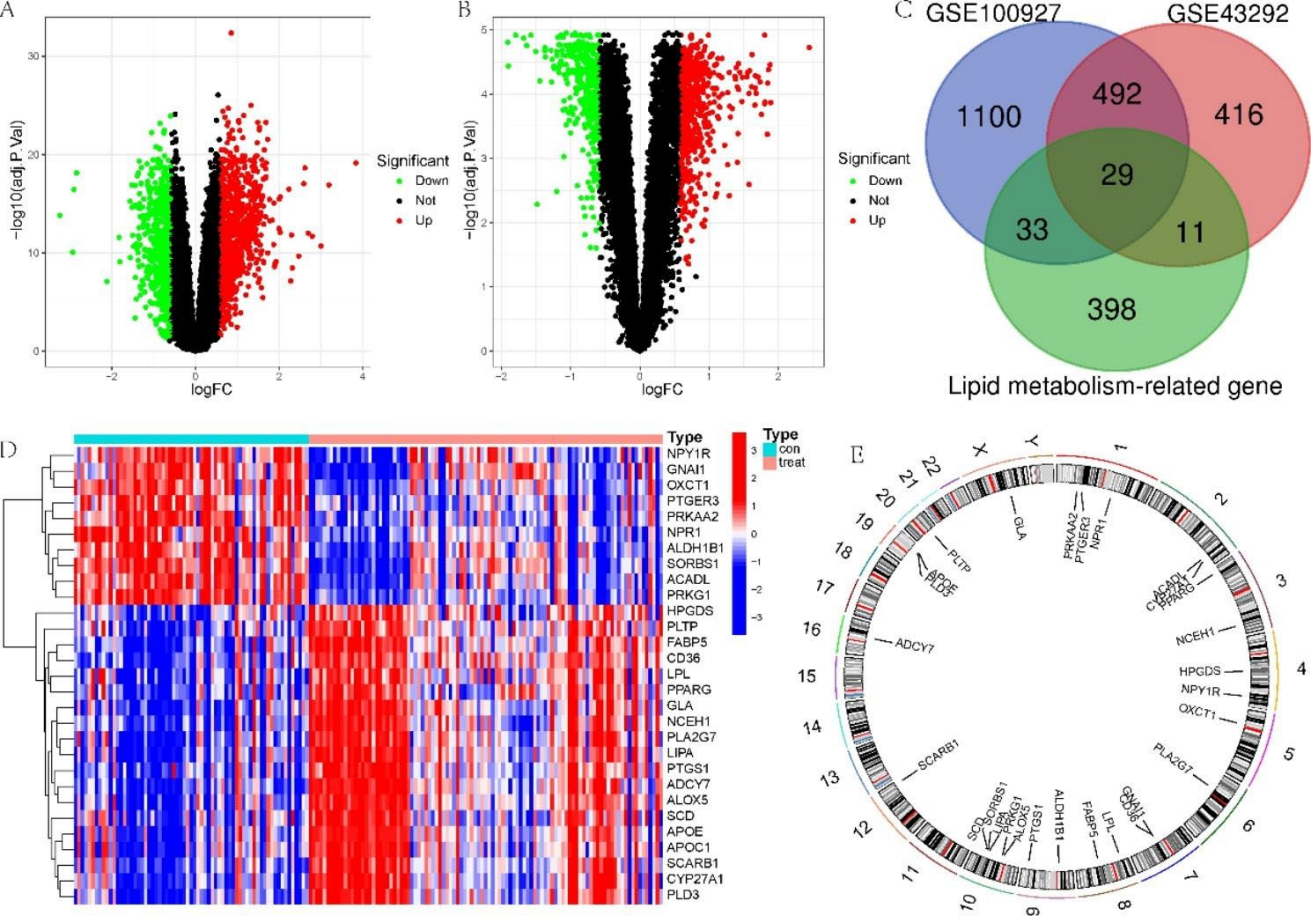
Differentially expressed LMRGs. **(A-B)** Volcano plot for differentially expressed genes in **(A)** GSE100927 and **(B)** GSE43292. **(C)** Venn diagram showing the 29 DE-LMRGs. **(D)** Heatmap showing 29 DE-LMRGs. **(E)** Location of LMRGs in chromosomes

The whole study process is depicted in Fig. 1. First, differential expression analysis was performed separately for the two AS datasets with adjusted *P* values less than 0.05 and |(log2 FC)| greater than 0.585 (Fig. 2A and B). The DEGs of the two datasets and the collected LMRGs were subsequently intersected to obtain a total of 29 DE-LMRGs (Fig. 2C). Among them, the expression levels of NPY1R, GNAI1, OXCT1, PTGER3, PRKAA2, NPR1, ALDH1B1, SORBS1, ACADL, and PRKG1 were lower, whereas HPGDS, PLTP, FABP5, CD36, LPL, PPARG, GLA, NCEH1, PLA2G7, LIPA, PTGS1, ADCY7, ALOX5, SCD, APOE, APOC1, SCARB1, CYP27A1, and PLD3 gene expression levels were higher in AS than in normal samples (Fig. 2D). Figure 2E shows the position of DE-LMRGs in the chromosome.

### Functional enrichment analysis

**Fig. 3:**
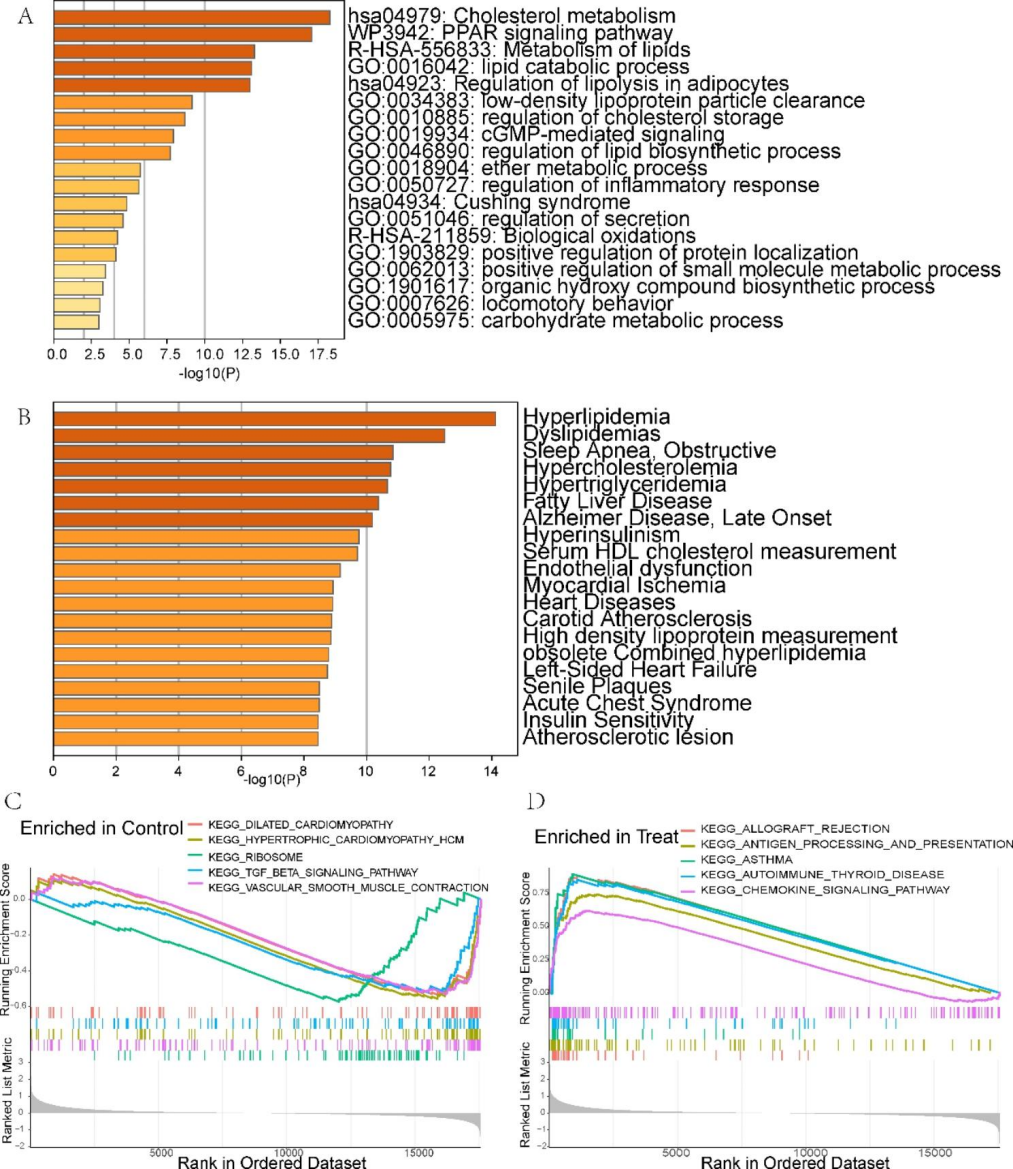
Functional enrichment analysis **(A)** Functional enrichment analysis. **(B)** DisGeNET enrichment analyses. (C-D) GSEA for **(C)** normal and **(D)** AS samples

To discover the potential functional relationship of these DE-LMRGs, we conducted a functional analysis of DE-LMRGs. PPI results showed that 29 genes were closely intertwined (Supplementary Fig. 1). The enrichment analysis of the Metascape results revealed marked enrichment in cholesterol and lipid metabolism, the PPAR signaling pathway, and regulation of the inflammatory response (Fig. 3A). Then, we found that these genes were strongly related to hyperlipidemia, hypercholesterolemia, and atherosclerotic lesions (Fig. 3B). All of the above results supported these 29 DE-LMRGs for AS discrimination. In addition, we utilized GSEA to investigate potential biological and functional differences between AS and normal samples (Fig. 3C and D).

### Identification of LMRG-related clusters in AS

**Fig. 4:**
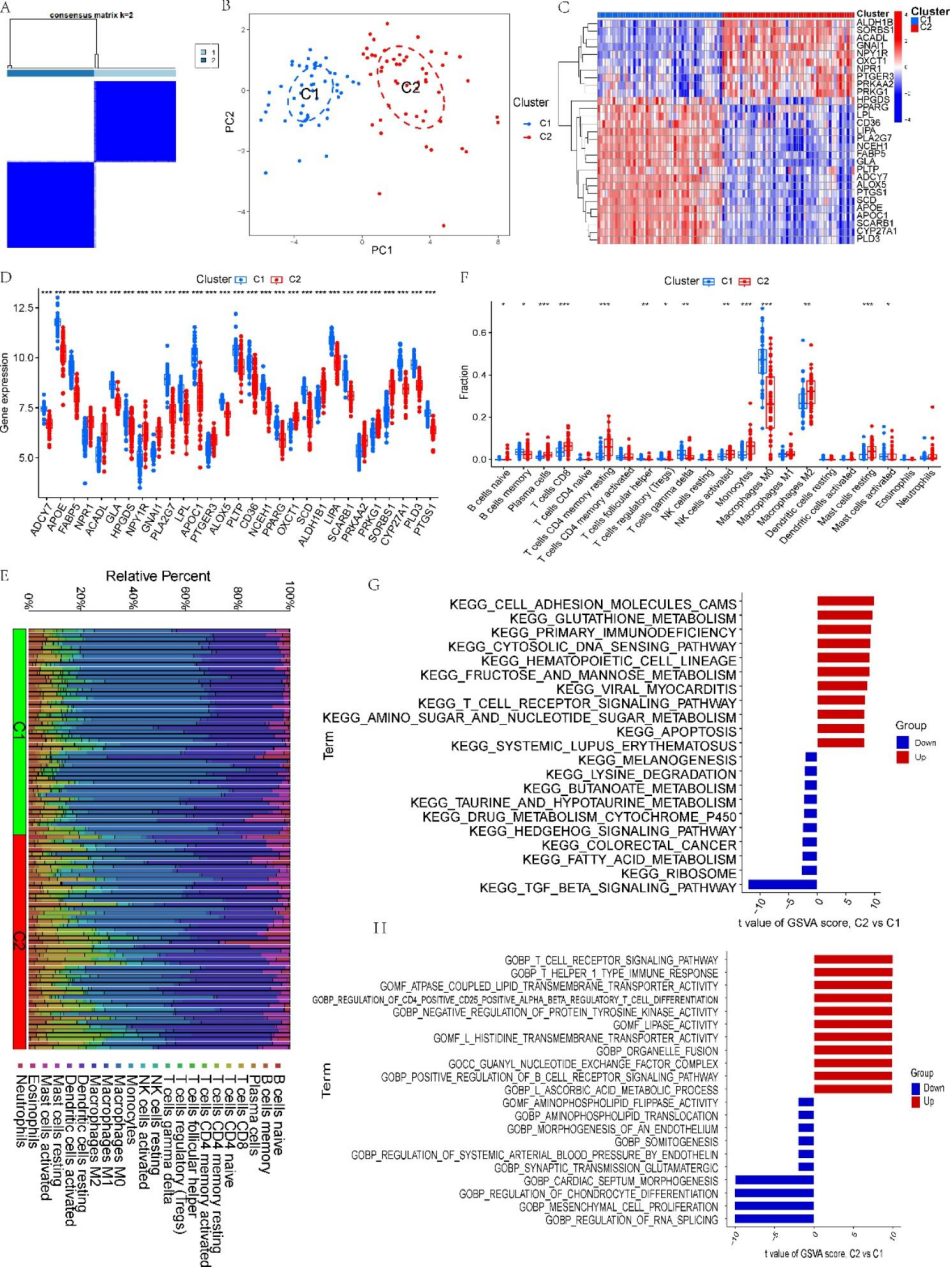
Identification of LMRG-related molecular clusters in AS. **(A)** Consensus clustering matrix with k = 2. **(B)** PCA shows subtype distribution. **(C)** The expression patterns of 29 DE-LMRGs in two clusters. **(D)** The expression of 29 DE-LMRGs was compared between two LMRG-related clusters using boxplots. **(E)** Heatmap of the immune infiltration profiles of the two clusters. **(F)** Comparison of immune cell infiltration between the two clusters. **(G-H)** GSVA analysis for **(G)** C1 and **(H)** C2 clusters. *P < 0.05; **P < 0.001; ***P < 0.0001

Using a consensus clustering technique, we categorized the AS samples according to 29 DE-LMRG expression profiles to clarify the LMRG-related expression patterns in AS. The results were most stable when divided into two clusters (Fig. 4A and Supplementary Fig. 2). PCA revealed a significant distinction between the two groups (Fig. 4B).

To analyze the molecular distinctions between clusters, we examined the expression differences of 29 LMRGs between C1 and C2. PPARG, HPGDS, CD36, LPL, LIPA, PLA2G7, NCEH1, FABP5, GLA, PLTP, ADCY7, ALOX5, PTGS1, SCD, APOE, APOC1, SCARB1, CYP27A1, and PLD3 were highly expressed in the C1 cluster, while ALDH1B1, SORBS1, ACADL, GNAI1, NPY1R, OXCT1, NPR1, PTGER3, PRKAA2, and PRKG1 were highly expressed in the C2 cluster (Fig. 4C and D). We then compared the differences in immune cell infiltration between the two clusters. We found that C1 had a significantly higher abundance of memory B cells, gamma delta T cells, M0 macrophages, and activated mast cells, and C2 had a significantly higher abundance of plasma cells, CD8 T cells, resting memory CD4 T cells, activated NK cells, monocytes, M2 macrophages, and resting mast cells (Fig. 4E F). Then, we utilized GSVA to investigate potential biological and functional differences between the two clusters. The results revealed that C2 was mainly enriched in some immune function-related pathways, while C1 was mainly involved in metabolism-related functions and pathways (Fig. 4G H). These results suggest that AS patients can be classified into two subgroups with significantly different biological functions, especially immune responses, based on the expression of LMRGs.

### Development and evaluation of the prognostic model

**Fig. 5:**
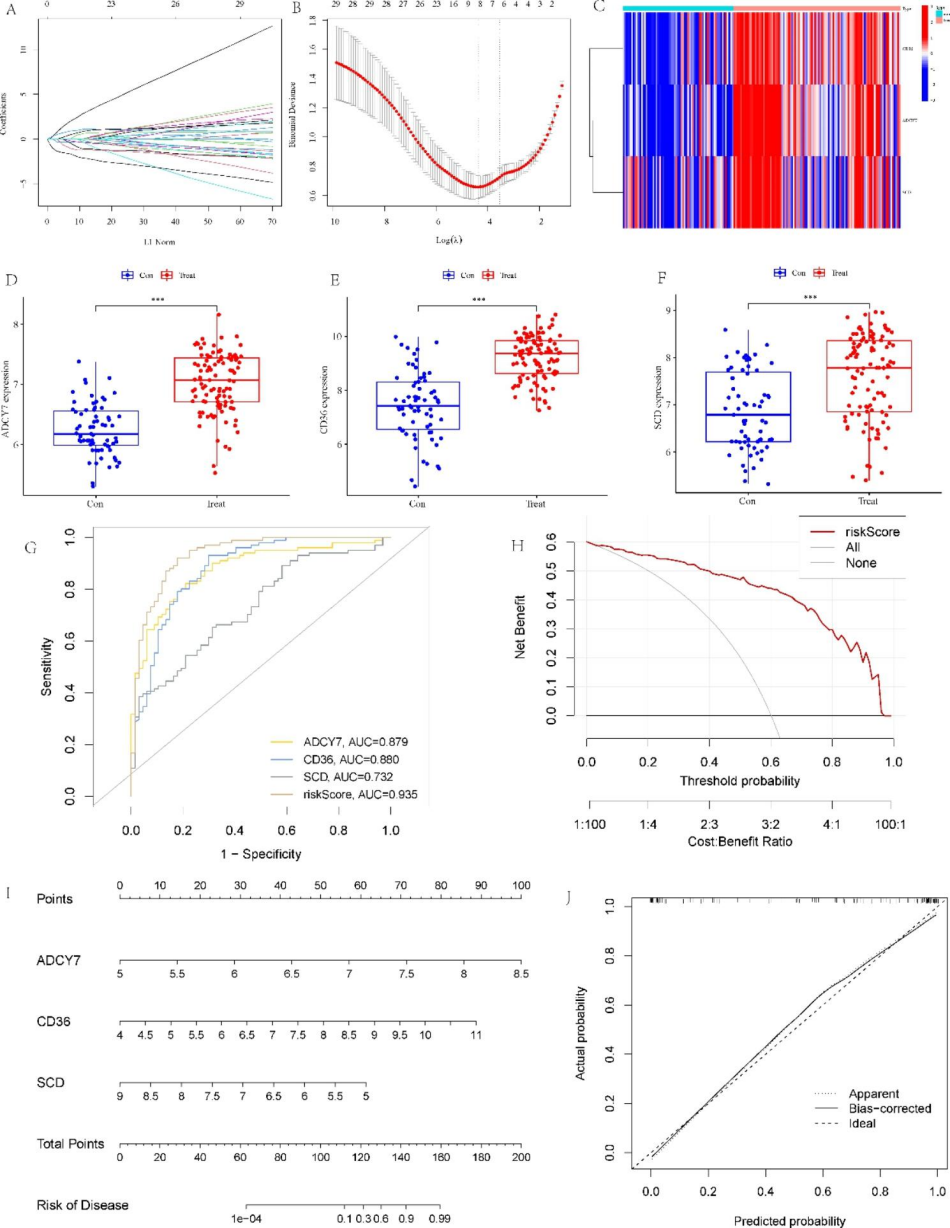
Establishment of the model using LASSO and logistic regression. **(A-B)** LASSO regression analysis with coefficient path diagram and cross-validation curve. **(C)** The heatmap depicts the levels of expression of the three LMRGs. **(D-F)** Box plots show the difference in **(D)** ADCY7, **(E)** CD36, and **(F)** SCD expression between AS and normal samples. **(G)** ROC analysis of the LMRG-related model. **(H)** Decision curve analysis of the predictive model. **(I)** Nomogram for forecasting AS risk. **(J)** Calibration curve to measure the model’s prediction ability. *P < 0.05; **P < 0.001; ***P < 0.0001

We then constructed AS prognostic models based on 29 DE-LMRGs. Given that formulas including too many variables could result in overfitting and that genes could exhibit colinearity, we reduced the candidate genes to minimize bias in this diagnostic model. These genes were screened by LASSO regression, and when log(λ) is -4.38 (based on lambda.min), the minimum deviation can be reached by relying on our model (Fig. 5A and B). Next, we analyzed these eight genes with logistic regression and found that only three key DE-LMRGs with a *P* value less than 0.05 were obtained: CD36, SCD, and ADCY7. As a result, the following formula was used to determine each patient’s risk score: Risk score = ADCY7 × (4.087018) + CD36 × (1.813650) + SCD × (− 2.193757). The heatmap shows the expression levels of the three model genes (Fig. 5C). The expression of the three model genes was significantly higher in AS samples than in normal tissues (Fig. 5D-F). Interestingly, we discovered that SCD protects against AS but is elevated, which seems to be in conflict. This may be because SCD is not a driver of AS and cannot directly cause pathogenesis, and its expression may be influenced by other genes. Meanwhile, we investigated the expression of gene mRNA. However, they ultimately function through the proteins they encode. Posttranslational modifications, epigenetics, negative feedback, and other factors can change the amounts of mRNA and proteins that are expressed.

In addition, we created an ROC curve to assess the diagnostic efficacy of the AS diagnostic model. The AUCs of the ROC curves of the risk score, ADCY7, CD36, and SCD were 0.935, 0.879, 0.880, and 0.732, respectively, demonstrating the model’s good predictive ability (Fig. 5G). The DCA curve showed that the clinical net benefit was higher when compared with the situation of either none or all for diagnosis (Fig. 5H). In addition, we developed a nomogram for estimating the risk of AS patients to further evaluate the prediction efficacy of the model (Fig. 5I). Next, the nomogram model’s prediction effectiveness was evaluated using the calibration curve. The calibration curve showed that the difference between the predicted risk of AS and the actual risk was minimal (Fig. 5J).

### Validation of the prognostic model in the test set

**Fig. 6:**
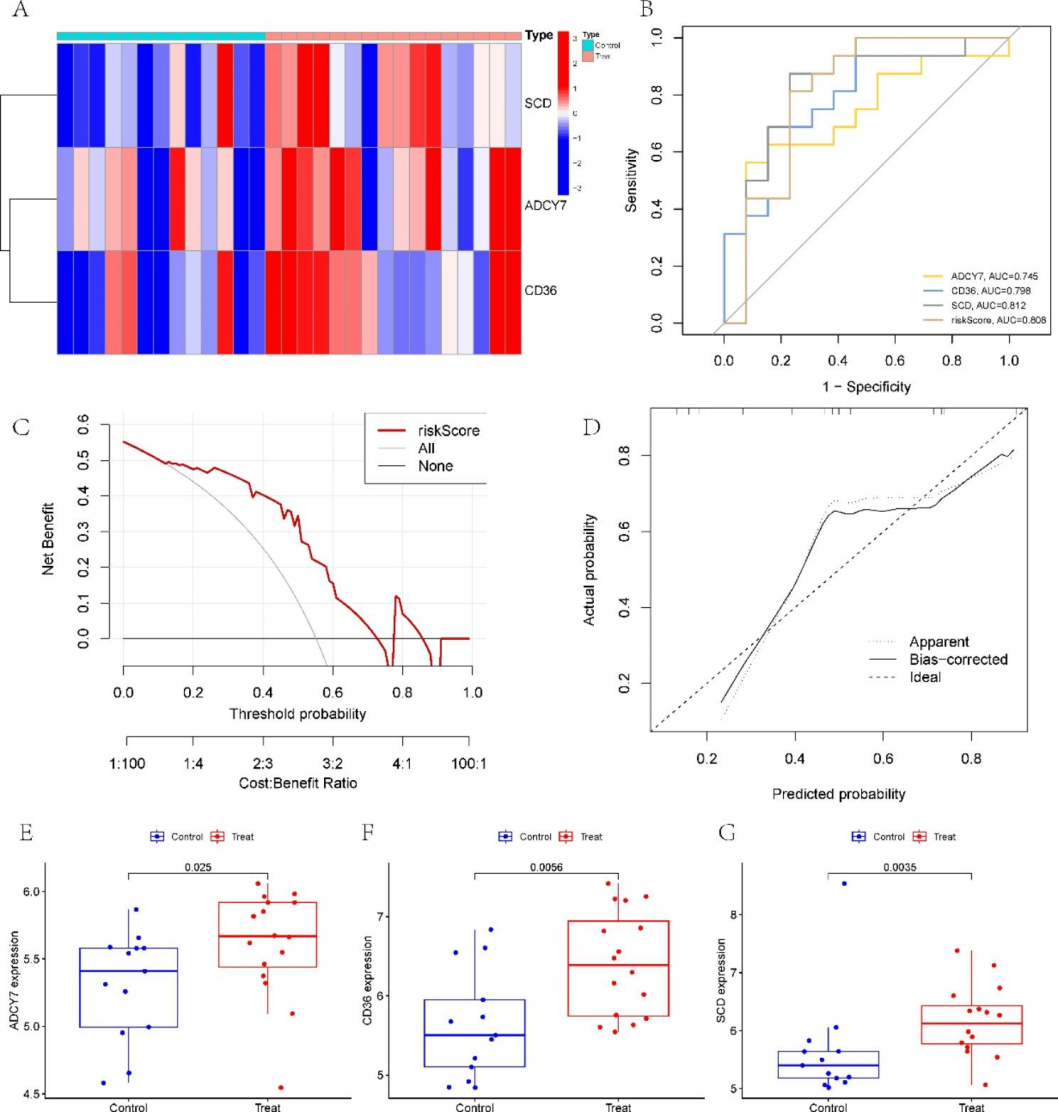
Validation of the LMRG-related model. **(A)** The heatmap depicts the levels of expression of the three LMRGs. **(B)** ROC analysis of the model in the test set. **(C-D)** Decision curve and calibration curve of the predictive model in the validation cohort. **(E-G)** Box plots show the difference in **(E)** ADCY7, **(F)** CD36, and **(G)** SCD expression between AS and normal samples in the test set. *P < 0.05; **P < 0.001; ***P < 0.0001

Then, using GSE28829, we verified our prediction model. The heatmap shows the expression levels of the three model genes in the test set (Fig. 6A). According to ROC curves, the three-LMRG risk model performed satisfactorily with an AUC value of 0.808 (Fig. 6B), demonstrating that our prediction model is effective in differentiating AS from normal people. The DCA and calibration curves also illustrated the good performance of our model (Fig. 6C and D). In addition, the three LMRGs were significantly highly expressed in the AS samples, consistent with the training set (Fig. 6E-G).

### GSVA and immune correlation analysis of three model LMRGs

**Fig. 7:**
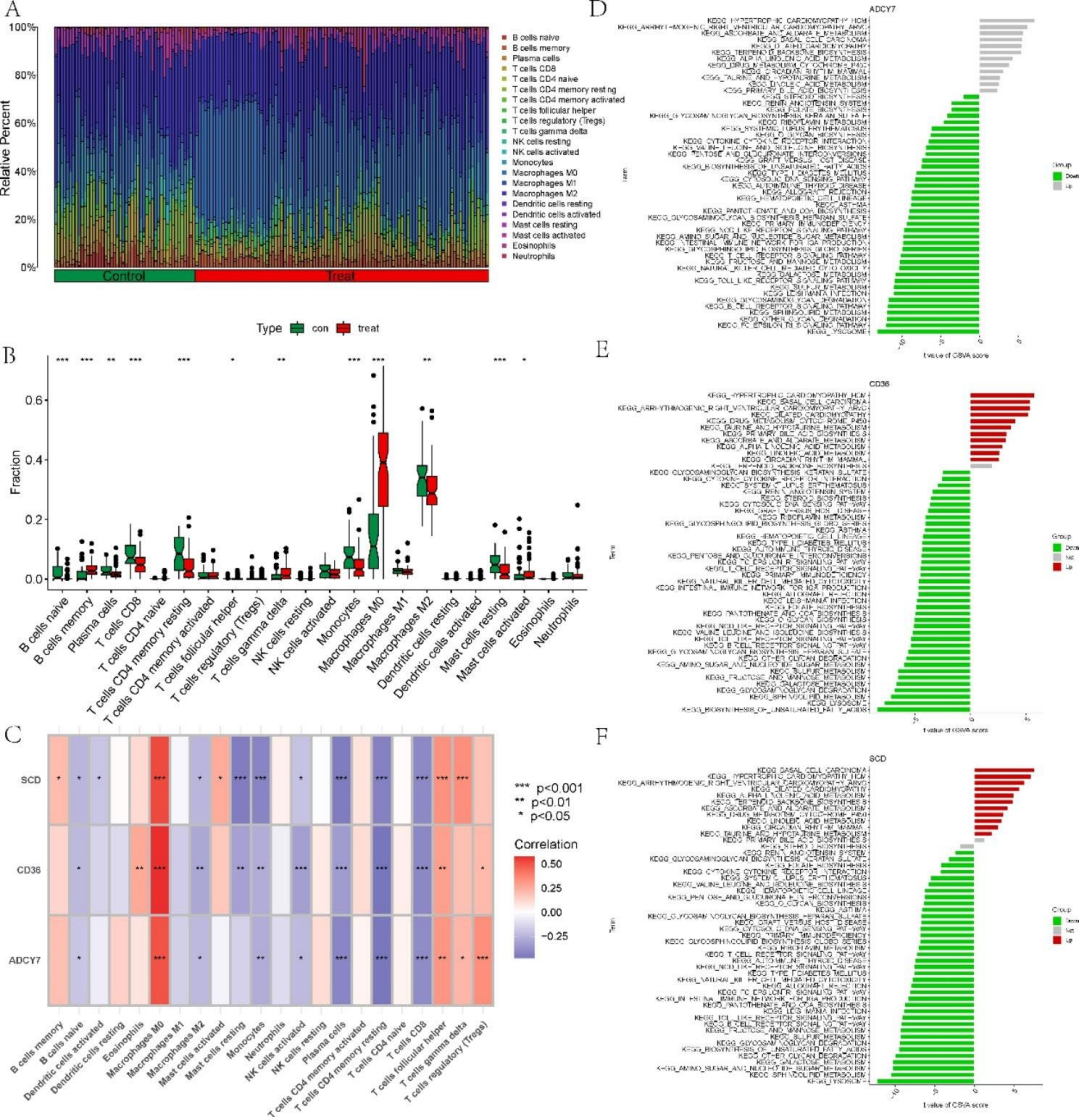
Immune cell correlation analysis and GSVA of model genes. **(A)** Immune infiltration profiles of AS and normal samples analyzed by CIBERSORT. **(B)** Comparison of immune cell infiltration between AS and normal samples. **(C)** Correlation between model genes and immune cells. **(D-F)** GSVA analysis of **(D)** ADCY7, **(E)** CD36, and **(F)** SCD. *P < 0.05; **P < 0.001; ***P < 0.0001

We first analyzed the differences in the abundance of immune infiltrates between AS and normal samples. The immune infiltration analysis showed a difference in immune cell types between AS and normal samples based on the CIBERSORT method. We found that plasma cells, CD8 T cells, resting memory CD4 T cells, monocytes, M2 macrophages, and resting mast cells were higher in normal samples. However, AS patients presented higher infiltration levels of memory B cells, gamma delta T cells, M0 macrophages, and activated mast cells, especially M0 macrophages, which were significantly higher than those in normal samples (Fig. 7A and B). Moreover, correlation analysis results indicated that a variety of immune cells, especially M0 macrophages, were significantly correlated with the three model LMRGs (Fig. 7C).

To further explore the potential mechanisms of the three model genes in AS, we used GSVA to analyze each model gene pathway enrichment difference. ADCY7 was mainly downregulated in some immune- and metabolism-related pathways (Fig. 7D). CD36 and SCD were mainly upregulated in some cardiomyopathy- and metabolism-related pathways and downregulated in some immune- and metabolism-related pathways (Fig. 7E F). The above findings revealed that the model LMRGs may be the key variables controlling the molecular and immune cell infiltration of AS.

### Single-cell data analysis and cell communication

**Fig. 8:**
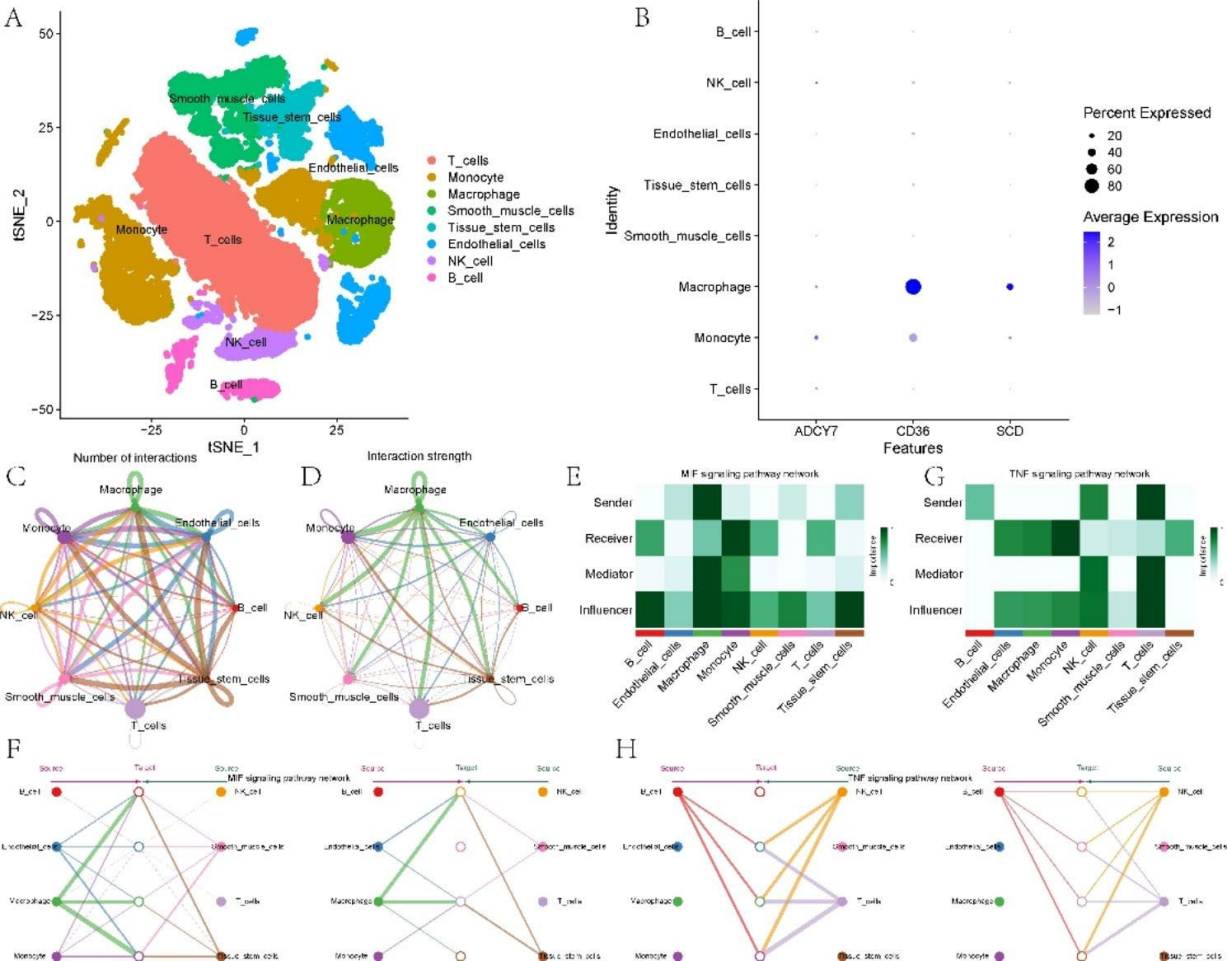
Single-cell analysis. **(A)** Cell types after annotation of all cells. **(B)** Expression of model genes in different cell types. **(C-D)** Cell communication analysis of the landscape. **(E-F)** Cellular communication analysis of MIF signaling. (G-H) Cellular communication analysis of TNF signaling

The previous analysis showed that three model genes were significantly associated with macrophages. Taking into account the important role of macrophages in the initiation and progression of AS. We next used scRNA-seq data for further analysis. Figure 8 A shows the annotation results of the scRNA-seq data. We subsequently found that the three model genes were mainly expressed in macrophages and monocytes. Figure 8 C and 8D demonstrate the interactions between different cell types. Macrophage migration inhibitory factor (MIF) is a pivotal mediator of atherosclerotic lesion formation[27]. The TNF signaling pathway is also closely associated with inflammation and AS[28]. Therefore, we further investigated MIF and TNF signaling between different cell types. For the MIF signaling pathway, macrophages were the main signal source cells, and monocytes were the main target cells (Fig. 8E-F). For the TNF signaling pathway, T cells and NK cells were the main signal source cells, and monocytes and macrophages were the main target cells (Fig. 8G-H).

## Discussion

AS is a major cause of morbidity and mortality worldwide, and the study of diagnostic signatures is important for the early diagnosis and high-quality treatment of AS[29]. The development and progression of AS, a chronic multifactorial disease, are correlated with an imbalance of pro- and anti-inflammatory factors against the backdrop of problems of lipid metabolism [30]. Lipid peroxidative stress and inflammatory responses caused by a variety of dangerous factors lead to structural damage to endothelial cells, which causes an inflammatory fibroproliferative response that impairs endothelial cell function [31]. It has also been suggested that AS is a process of lipid accumulation and lipid peroxidative stress, which involves a large number of lipid peroxidative stress factors [32]. Therefore, deciphering the mechanisms by which lipid metabolism triggers AS is essential for developing new therapeutic strategies to reduce the burden of AS due to the perturbation of lipid metabolism. Transcriptomes are useful and practical tools for studying metabolism-related issues, although it is difficult to measure and study them in a direct manner. In the present research, we identified novel LMRG-related molecular subtypes and diagnostic signatures in AS and explored their function in disease diagnosis and association with immune characteristics. These results reflect that the reprogramming of lipid metabolism in AS not only affects disease progression but is also involved in the remodeling of the immune population.

In this study, we first identified 29 AS-specific LMRGs by differential expression analysis. We found by enrichment analysis that AS-specific LMRGs are involved in cholesterol and lipid metabolism, the PPAR signaling pathway, and inflammatory response regulation and are also closely associated with atherosclerotic lesions, indicating the prominent biological significance of these genes in the pathogenesis of AS. Furthermore, two separate LMRG-related clusters were found using unsupervised cluster analysis to highlight the various LMRG-related patterns in AS patients according to the expression landscapes of LMRGs. AS represents a systemic chronic inflammatory disease involving an activated innate immune response [33]. AS lesions are filled with immune cells that may coordinate and trigger the inflammatory response [34]. There is growing evidence of immune diversity inherent in AS plaques [35]. In addition, immune cell dysfunction, such as abnormal distribution of abundance and type, contributes to AS progression [36]. An in-depth exploration of the immune infiltration of different clusters may help to better understand the progression of AS. We found that the two clusters showed significantly different biological functions, especially in the immune response, and C2 had a significantly higher abundance of immune cell infiltration than C1.

Subsequently, by LASSO and logistic regression, we developed a diagnostic model based on three LMRGs (ADCY7, CD36, and SCD). The AUC values of the ROC and DCA curves showed good predictive power. Additionally, in the external validation set, the three model genes showed expression trends consistent with those in the training set. Importantly, the AUC values of the validation set also showed good predictive ability of the model. Adenylate cyclase 7 (ADCY7) encodes a membrane protein that is capable of participating in extracellular signaling in intracellular responses [37]. Studies have found that macrophages derived from ADCY7-deficient mice produce more of the proinflammatory cytokine TNF-α [38]. However, to the best of our knowledge, the role of ADCY7 in the pathogenesis of AS has not been summarized. CD36 is a type 2 cell surface scavenger receptor that is widely expressed in many immune and nonimmune cells [39]. CD36 receptors can enter macrophages and cause them to form foam cells, and blocking foam cell formation can be achieved by decreasing CD36 expression[40, 41]. Additionally, some studies have reported that CD36 deficiency prevents the development of AS [42]. Stearoyl-CoA desaturase (SCD) is a central enzyme in lipid metabolism for the synthesis of monounsaturated fatty acids and a key control gene associated with AS. SCD was found to protect human arterial endothelial cells from lipotoxicity [43].

To further understand the potential mechanisms of model genes in AS, we explored the association of model genes with immune cells. We found that three model genes were closely associated with a variety of immune cells, especially M0 macrophages. Subsequent GSVA analysis also revealed a close association between model genes and immune-related pathways. In addition, we further identified three LMRGs that were predominantly expressed in macrophages in the single-cell data dimension. In summary, LMRGs may contribute to disease onset and progression by affecting the immune microenvironment in AS, especially macrophages.

## Study strengths and limitations

To the best of our knowledge, our study is the first to use bioinformatics to determine the involvement of LMRG in AS. However, we retrospectively analyzed data obtained using publicly available databases. Given the rigor of our study and the novelty of the prognostic model, our results should be validated in prospective multicenter studies. Moreover, additional experimental studies are needed to uncover the underlying mechanism of the correlation between lipid metabolism and AS development.

## Conclusion

In conclusion, our study revealed the link between LMRGs and infiltrated immune cells as well as the great heterogeneity of immunological cells between AS patients with different clusters. Meanwhile, we developed the first LMRG-based diagnostic model, which will aid in the clinical diagnosis and treatment of AS in the future. Our study is helpful to better understand the pathogenesis and progression of AS and provides a theoretical basis for future studies on lipid metabolism in AS.

## Electronic supplementary material

Below is the link to the electronic supplementary material.

Supplementary Fig. 1. Gene relationship network diagram among the DE-LMRGs.

Supplementary Fig. 2. Identification of LMRG-related molecular clusters in AS.

## Data Availability

The datasets analyzed in this work can be retrieved from GEO databases. The relevant code can be obtained from the following URL (https://github.com/pan-xue/local/blob/main/AS.R). Additionally, any analytic technology-related questions can be directly contacted by the corresponding author.
