# Supplementary figures and images for: Identification of lipid metabolism-related biomarkers for diagnosis and molecular classification of atherosclerosis
Source: Lipids Health Dis. 2023 Jul 6;22:96. doi: 10.1186/s12944-023-01864-6 (PMC10324206; doi:10.1186/s12944-023-01864-6)

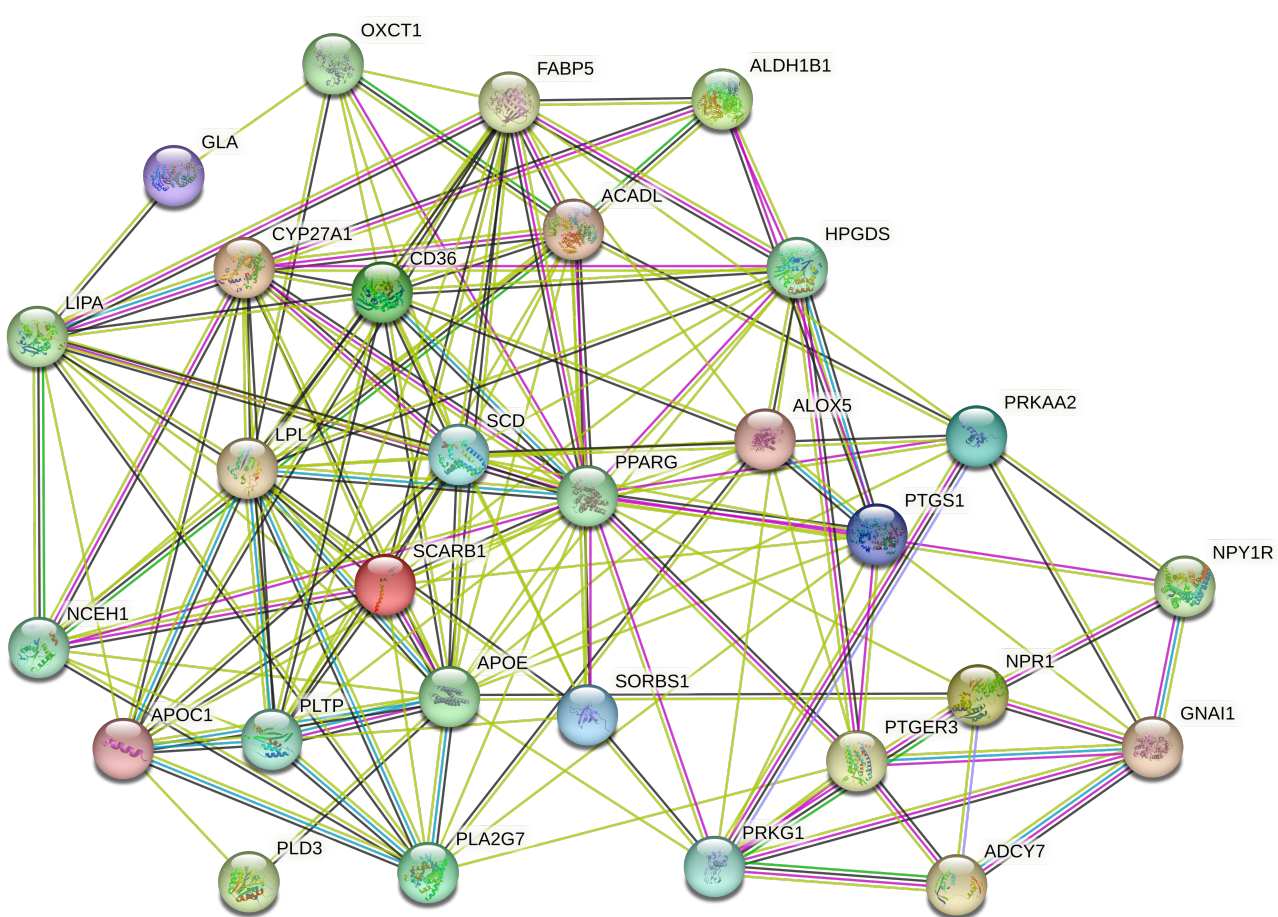

Supplement: Supplementary file 1 — Supplementary Fig. 1. Gene relationship network diagram among the DE-LMRGs. [file 12944_2023_1864_MOESM1_ESM.pdf]

consensus CDF

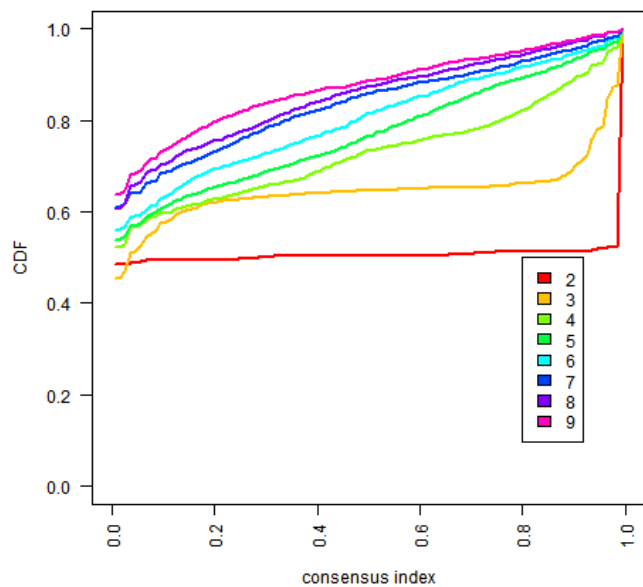

Delta area

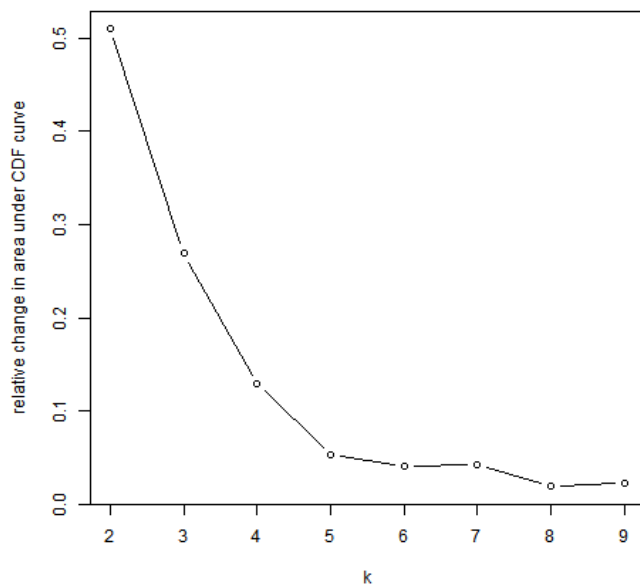

tracking plot

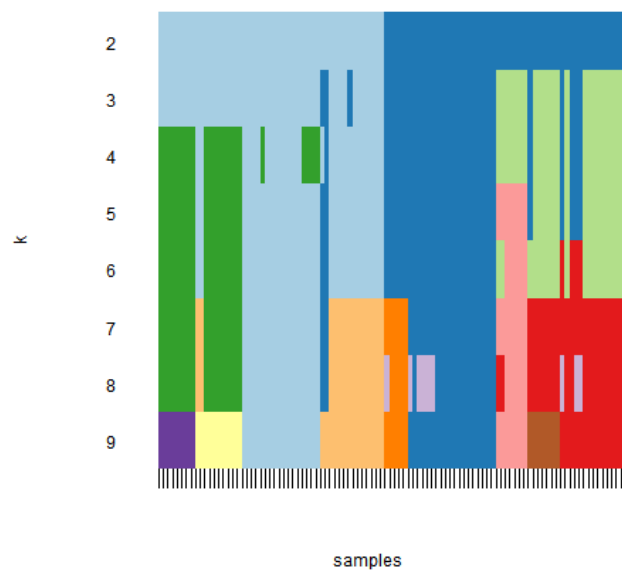

Supplement: Supplementary file 2 — Supplementary Fig. 2. Identification of LMRG-related molecular clusters in AS. [file 12944_2023_1864_MOESM2_ESM.pdf]
